# Chromosome mapping of repetitive sequences in Anostomidae species: implications for genomic and sex chromosome evolution

**DOI:** 10.1186/1755-8166-5-45

**Published:** 2012-12-11

**Authors:** Edson Lourenço da Silva, Rafael Splendore de Borba, Patrícia Pasquali Parise-Maltempi

**Affiliations:** 1 Departamento de Biologia, Laboratório de Citogenética, Instituto de Biociências, Universidade Estadual Paulista “Julio de Mesquita Filho” - UNESP, Av. 24A, 1515, Rio Claro, SP, CEP 13506-900, Brazil

**Keywords:** Chromosomes, FISH, Heterochromatin, Neotropical fish, Sex chromosomes

## Abstract

**Background:**

Members of the Anostomidae family provide an interesting model system for the study of the influence of repetitive elements on genome composition, mainly because they possess numerous heterochromatic segments and a peculiar system of female heterogamety that is restricted to a few species of the *Leporinus* genus. The aim of this study was to isolate and identify important new repetitive DNA elements in Anostomidae through restriction enzyme digestion, followed by cloning, characterisation and chromosome mapping of this fragment. To identify repetitive elements in other *Leporinus* species and expand on studies of repetitive elements in Anostomidae, hybridisation experiments were also performed using previously described probes of Le*Spe*I repetitive elements.

**Results:**

The 628-base pair (bp) Le*Spe*II fragment was hybridised to metaphase cells of *L. elongatus* individuals as well as those of *L. macrocephalus*, *L. obtusidens*, *L. striatus*, *L. lacustris*, *L. friderici*, *Schizodon borellii* and *S. isognathus*. In *L. elongatus*, both male and female cells contained small clusters of Le*Spe*II repetitive elements dispersed on all of the chromosomes, with enrichment near most of the terminal portions of the chromosomes. In the female sex chromosomes of *L. elongatus* (Z_2_,Z_2_/W_1_W_2_), however, this repeated element was absent. In the remaining species, a dispersed pattern of hybridisation was observed on all chromosomes irrespective of whether or not they were sex chromosomes. The repetitive element Le*Spe*I produced positive hybridisations signals only in *L. elongatus*, *L. macrocephalus* and *L. obtusidens*, i.e., species with differentiated sex chromosomes. In the remaining species, the Le*Spe*I element did not produce hybridisation signals.

**Conclusions:**

Results are discussed in terms of the effects of repetitive sequences on the differentiation of the Anostomidae genome, especially with respect to sex chromosome evolution. Le*Spe*II showed hybridisation patterns typical of Long Interspersed Elements (LINEs). The differential distribution of this element may be linked to sex chromosome differentiation in *L. elongatus* species. The relationship between sex chromosome specificity and the Le*Spe*I element is confirmed in the species *L. elongatus*, *L. macrocephalus* and *L. obtusidens*.

## Background

Studies of Neotropical fishes indicate that only a few numbers contain heteromorphic sex chromosomes [1]. However, simple and multiple systems of heterogamety have been identified in various groups of fish [2-4]. An interesting feature in the distribution of these systems is that for both male and female heterogamety, multiple and simple systems, as well the sporadic occurrence of heterogamety, can be found in related species and in different populations of the same species [2]. This diversity in the sex chromosome structure of fishes has been attributed in part to the dynamics of repetitive DNA present on the chromosomes [5-8].

In the eukaryotic genome, there are two classes of repetitive elements: sequences organised in tandem repeats such as satellites, minisatellites and microsatellites, and sequences dispersed in the genome as transposons and retrotransposons [9]. In eukaryotes, protein-coding sequences can constitute as little as 2–10% of the genome, with the remainder of the genome comprising introns, intragenic regions, regulatory DNA sequences and a variety of repetitive sequences [10,11].

Members of the Anostomidae family, particularly the *Leporinus* genus, are an interesting model system for the study of repetitive elements that influence the composition of the genome. This genus is particularly attractive due to the presence of the peculiar sex chromosome system ZZ and ZW in some species. Some studies focusing on the isolation, characterisation and correlation of these repetitive elements with the presence and evolution of sex chromosomes have highlighted the great potential of studying these elements in these Anostomidae species [5,12-15].

The repetitive element Le*Spe*I, which was previously isolated from the species *L. elongatus*[5], shows a sex-specific pattern that highlights the relationship of this element to the process of sex chromosome differentiation in *L. elongatus*, as well the congeneric species *L. macrocephalus* and *L. obtusidens*[13]. Besides this sequence was not found in *L. friderici* (which lack differentiated sex chromosomes), reinforcing the notion that this element is linked to sex chromosome differentiation [13]. In addition, Le*Sma*I, another satellite DNA element isolated from *L. elongatus*, displayed the species-specific characteristic of localisation near the nucleolar organiser region in both males and females [14]. Interestingly, at least in *L. elongatus*, the nucleolar organiser region-bearing chromosomes may comprise a probable multiple sex chromosome system (Z_2_ and W_2_).

Indeed, the participation of repetitive sequences in sex chromosome differentiation in Anostomidae is certain; however, the role of these sequences in this complex process is still unknown. In this study, we identified a new repetitive element from *L. elongatus*, Le*Spe*II. In addition, we investigated the localisation of the repetitive element Le*Spe*I in four Anostomidae species not studied previously to help elucidate the association of repetitive sequences in the diversification of the Anostomidae genome, particularly sex chromosome differentiation.

## Results

### Chromosome number of anostomids and analysis of a repetitive sequence

**Figure 1:**
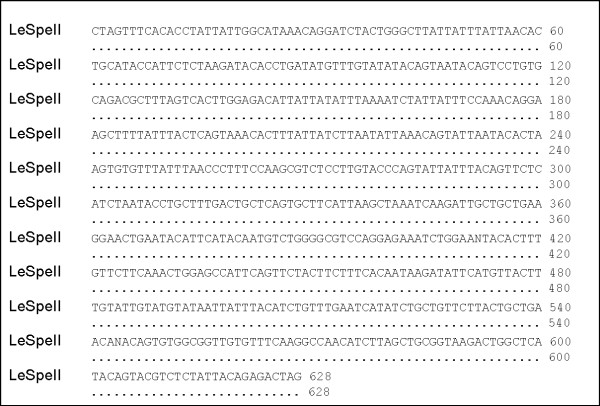
Sequence of Le*Spe*II clone.

Cytogenetic analysis of various anostomids revealed a diploid chromosome number of 54, with both metacentric- and submetacentric-type chromosomes. The cloned fragments (628 bp) had high AT content (64.9%) and showed perfect sequence alignment among the clones (Figure 1). A BLASTN search did not reveal significant similarities between this sequence and previously identified sequences. This newly identified sequence was designated Le*Spe*II.

### Genomic hybridisation of Le*Spe*II

**Figure 2:**
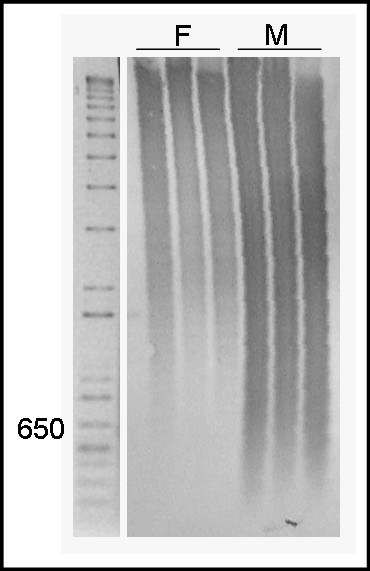
Southern blot of DNA digested with SpeI, using the Le*Spe*II sequence as probe in (F) female and (M) male *L. elongatus *individuals.

The Le*Spe*II probe was labelled and hybridised to the digested genomic DNA of male and female *L. elongatus*, individuals using Southern blot analysis. The probe hybridised to many different restriction fragments of various sizes, rather than to one band, which indicates that Le*Spe*II is predominately dispersed, not tandemly arrayed (Figure 2).

### Chromosomal distribution of repetitive DNA

**Figure 3:**
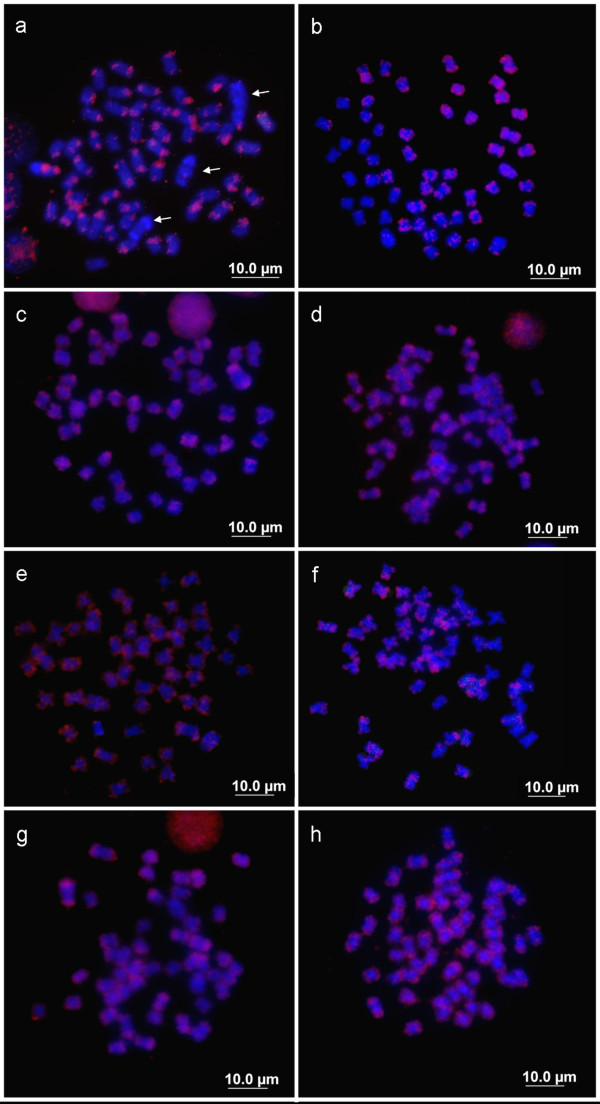
**Mitotic metaphase chromosomes of Anostomidae representatives hybridised with the Le*Spe*II repetitive element.a**- *Leporinus elongatus* (female); **b**- *L. macrocephalus* (male); **c**- *L. obtusidens* (female); **d**- *L. friderici* (female); **e**- *L. striatus* (female); **f**- *L. lacustris* (female); **g**- *Schizodon borellii* (female); **h**- *S*. *isognathus*. Arrows indicate sex chromosomes in *L. elongatus.*

The chromosomal distribution of the Le*Spe*II element was determined by Fluorescence in situ hybridisation (FISH) analysis using metaphase spreads of male and female *L. elongatus* individuals. Small clusters of signals were found to be dispersed throughout all of the chromosomes, with enrichment near most terminal portions of the chromosomes (Figure 3a). In the sex chromosomes of *L. elongatus*, however, this repeated element was absent, even when low stringency wash conditions were employed (Figure 3a). In the remaining species, i.e., *L. macrocephalus*, *L. obtusidens L. friderici, L. striatus, L. lacustris*, *Schizodon borellii* and *S. isognathus*, a dispersed signal pattern was observed in all chromosomes, with no indication of sex specificity (Figure 3b–h).

**Figure 4:**
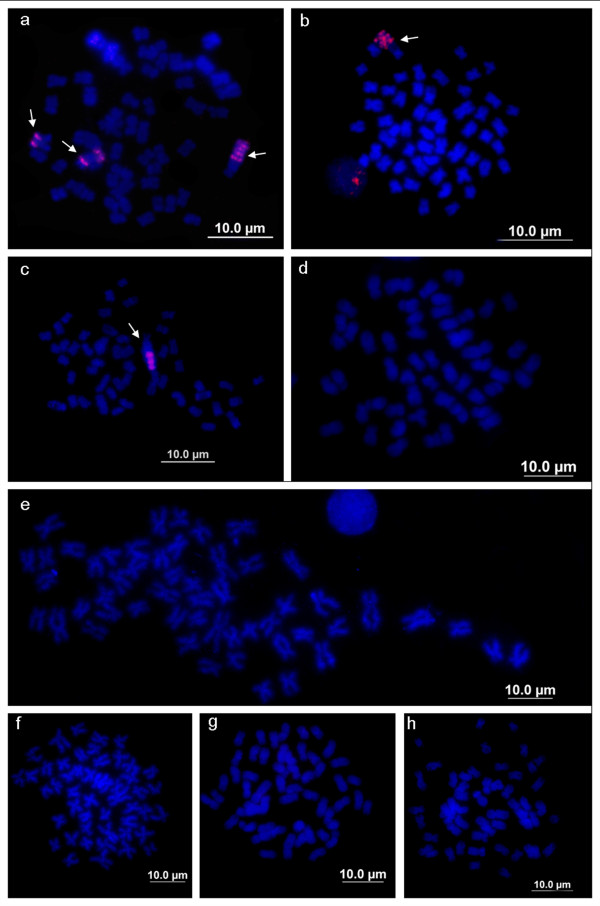
**Mitotic metaphase chromosomes of female Anostomidae representatives hybridised with Le*Spe*I repetitive element.a**- *Leporinus elongatus*; **b**- *L. macrocephalus*; **c**- *L. obtusidens*; **d**- *L. friderici*; **e**- *L. striatus*; **f**- *L. lacustris*; **g**- *Schizodon borellii*; **h**- *S isognathus*. Arrows indicate sex chromosomes in *L. elongatus, L. macrocephalus* and *L. obtusidens.*

The distribution patterns of the Le*Spe*I element in *L. elongatus, L. macrocephalus*, *L. obtusidens* and *L. friderici* were similar to those described by Parise-Maltempi et al. [4] and Marreta et al. [16], who detected positive signals on the Z_2_Z_2_ chromosomes of males and the W_1_Z_2_ and W_2_ chromosomes of female individuals of *L. elongatus* (Figure 4a); while in *L. macrocephalus* and *L. obtusidens* females, positive signals were detected on the long arm of the W chromosome (Figure 4b,c). *L. friderici, L. striatus, L. lacustris*, *Schizodon borellii* and *S. isognathus* did not show Le*Spe*I hybridisations signals even under low stringency conditions (Figure 4d–h).

## Discussion

Several repetitive elements have previously been isolated from several *Leporinus* species. These elements exhibited wide diversity with respect to chromosomal location and distribution [5,12-15]. The organisational patterns of these repetitive sequences demonstrate the dynamics of the fish genome, despite the apparent chromosomal stability that has been observed in the Anostomidae family since the first cytogenetic studies were performed in the 1980s (E.L. Silva, personal communication).

The predominantly dispersed distribution pattern of the Le*Spe*II element revealed by Southern blot analysis was consistent with the genomic organisation of most, if not all, previously analysed LINEs. Moreover, this characteristic distribution pattern differed from that of other previously described repetitive elements of the anostomids. LINE elements comprise a class of Non-LTR retrotransposons, which are an important group of repetitive DNA elements widely studied in many organisms (see [6]). The accumulation of Le*Spe*II near the telomeres of almost all chromosomes of the analysed anostomids was similar to the distribution pattern observed for the LINE CiLINE2 [17]. Higashiyama et al. [18] suggested that because these elements display terminal distribution, they may play a role in the stabilisation of the chromosomes on which they reside, in some circumstances, or they might form a structural cap to protect the terminal portions of the chromosomes. In fact, the sex chromosome system of *L. elongatus* is considered to still be undergoing evolution [5]; thus the absence of Le*Spe*II favours an environment of instability, which is required for ongoing evolution.

In some groups of vertebrates, dispersed elements are arranged in clusters and blocks that can easily be visualised on chromosomes [19,20], but in some fish species, these elements have a widely scattered distribution pattern on all of the chromosomes [19,21]. These sequences have been associated with the evolution of the genome size of the host organism [22]. Indeed, these sequences elicit alterations in gene function through the process of insertion, inducing chromosomal rearrangements and the production of coding and non-coding material, thereby allowing new genes or new regulatory sequences to emerge [23]. These elements are therefore potentially associated with speciation events [24].

A series of repetitive DNA elements isolated from the *Leporinus* genus have been used as probes in chromosome mapping. All of these DNA families presented different distribution patterns, adding unique features to some species, including sex-specific sequences and species-specific repeated elements. Nakayama et al. [12] described two different sequences, including one found in both Z and W chromosomes and the other representing a second family specific to the W chromosome. Le*Spe*I repetitive DNA was described as a sex-specific dispersed repetitive element with distinct distribution patterns on two exclusive female chromosomes, named W_1_ and W_2_ by Parise-Maltempi et al. [5]. This sequence was used to probe chromosomal preparations from *L. macrocephalus, L. obtusidens* and *L. friderici*, which displayed positive signals only on the W chromosomes of *L. macrocephalus* and *L. obtusidens*[13]. Here, we examined the distribution patterns of Le*Spe*I in these species, as well in *L. striatus*, *L. lacustris*, *Schizodon borellii* and *S. isognathus*, which are all from different hydrographic basins, and we corroborated the sex chromosome specificities of this sequence. Besides the repetitive sequences associated with differentiated sex chromosomes, also a satellite DNA, Le*Sma*I, has also been isolated from *L. elongatus*[14]*.* This satellite DNA, which is linked to nucleolar organiser regions (classified as chromosomes Z_2_ and W_2_ by Parise-Maltempi et al. [5]), is exclusively found in *L. elongatus*, in both male and female individuals.

The simple sex chromosome system originated from a pair of autosomes that, for some reason, stopped recombining and gradually diverged from each other [25]. This process of sex-chromosome evolution has attracted considerable interest over the years, and an important question has been what the evolutionary forces are that act to make a pair of autosomes cease recombining in one sex, eventually leading to the formation of two discrete chromosome types [26].

A series of heterochromatin acquisitions may have driven the morphological differentiation of simple sex chromosomes found in some *Leporinus* species to the multiple sex chromosome system observed in *L. elongatus*. Considering the direct relationship between the Le*Spe*I element and sex chromosome differentiation, at least in *L. elongatus*, *L. macrocephalus and L. obtusidens*, the presence of Le*Spe*II in all of the anostomids studied here, including those without differentiated sex chromosomes (such as *Leporinus friderici*, *L. striatus*, *L. lacustris*, *Schizodon borellii* and *S. isognathus*), may represent an acquisition that is more ancient than that of the remaining repetitive elements already described in this family.

In the *L. elongatus* genome, the Le*Spe*II element is absent from the sex chromosomes (W_1_, Z_2_ and W_2_), but it is widely distributed in the remaining autosomes. The absence of this element on W_1_, Z_2_ and W_2_ may have contributed to the fixing of the other repetitive elements. During another stage of differentiation, the accumulation of Le*Spe*I and Le*Sma*I sequences may have modified the accumulation of repetitive sequences in the *L. elongatus* genome [5,14], and a remarkable event such as a rearrangement may have promoted the formation of a multiple sex system in this species, since another heteromorphic pair (Z_2_ and W_2_) was found only in this species [13].

In general, the acquisition of heterochromatin, which resulted in the development of a series of important characteristics unique to anostomids, is the most prominent route for chromosomal differentiation in this family [27-29]. Our results revealed amazing new characteristics of the Anostomidae genome and the results of the present study provide new insights into the pathways of chromosomal diversification of the species in this family and will be useful for future comparative genomic studies. The fishes, in general, comprise a basal group of vertebrates with a wide distribution of sex chromosome types that were brought about by events that resulted among others alterations, in changes in or multiplication of repetitive sequences. Therefore, this study also provides important insights into the evolution of the vertebrate genome.

## Materials and methods

### Chromosomal and genomic DNA preparation

Wild specimens of *Leporinus elongatus* (five males and eight females) were collected in the Mogi-Guaçú River, Pirassununga, São Paulo state, Brazil. Mitotic chromosome isolation and chromosome staining were performed according to Foresti et al. (1993) [30]. Additional cytogenetic studies were carried out with the species *L. macrocephalus* Garavello and Britiski 1988 (two males and two females), *L. obtusidens* (two males and two females), *L. striatus* Kner 1858 (one male and three females); *L. lacustris* Amaral Campos 1945 (one male and three females), *L. friderici* (Bloch 1794) (two males and three females), *Schizodon borellii* (Boulenger 1900) (two males and two females) and *S. isognathus* Kner 1858 (one male and one female), all of which were collected in the Paraguay River basin, Mato Grosso State, Brazil. Genomic DNA was extracted from liver and blood using standard methods [31]. The specimens were collected in accordance with collection license issued by Instituto Brasileiro do Meio Ambiente e dos Recursos Naturais Renováveis (19833-1), and the material was processed according to Colégio Brasileiro de Experimentação Animal (016/04 – CEEA).

The search for repetitive DNA was conducted using restriction enzyme digestions of genomic DNA of *L. elongatus* with different restriction endonucleases. The endonuclease *Spe*I produced a conspicuous band of approximately 650 bp. This DNA band was isolated from a gel, cloned into the pMOS Blue plasmid vector (Amersham Biosciences), and used for transformation in *E. coli* DH5α competent cells. Positive clones were identified and stored at −75°C for future analysis.

### Sequencing and sequence analysis

Two positives clones were isolated and sequenced using DYEnamic™ ET Terminator Cycle Sequencing (Amersham Bioscience) and an ABI 377 automated DNA sequencer (Applied Biosystems). Nucleotide sequences were subjected to a search at the National Center for Biotechnology Information (NCBI) (http://www.ncbi.nlm.nih.gov/blast) [32], and sequence alignment was performed using Clustal W [33] and manually checked.

### Genomic organisation

The genomic organisation of the isolated repetitive fragment was determined by Southern blot hybridisation with 10 μg aliquots of genomic DNA from three *L. elongatus* males and three females. Genomic DNA was digested with the restriction enzyme *Spe*I, and the restriction fragments were separated by electrophoresis in a 1.5% agarose gel and transferred onto a Hybond N+ nylon membrane (Amersham Bioscience) by capillary action. Clones bearing the isolated repetitive fragment were used as probes and hybridised under high stringency conditions using an ECL Direct Nucleic Acid Labelling and Detection Systems Kit (Amersham Biosciences), according to the manufacturer’s instructions.

### Chromosome mapping

Chromosome mapping of repetitive sequences was performed using the isolated fragment and probes of the Le*Spe*I repetitive element [5] through FISH, according to the method of Pinkel et al. [34] with modifications described in Silva et al. (2012) [14]. The probes were labelled with digoxigenin in a second cycle of reamplification using the clone as the template for PCR with an M13 primer set (F 5’-TGT AAA ACG ACG GCC AGT-3^′^; R 5’-CAG GAA ACA GCT ATG ACC-3’). Hybridisation signals were detected using appropriate antibody sets composed of antidigoxigenin-rhodamine to detect the signals from the hapten digoxigenin.

Chromosomes were counterstained with DAPI mounted with antifade solution and observed using an Olympus BX51 microscope coupled to an Olympus digital camera (model D71). Chromosome images were captured using the DP Controller program.

## Abbreviations

BLASTN: Basic Local Alignment Search Tool; DAPI: 4, 6 diamino-2-phenylindole; FISH: Fluorescence in situ hybridisation; LINEs: Long Interspersed Elements; LTR: Long Terminal Repeat; PCR: Polymerase Chain Reaction.

## Competing interests

The authors declare that they have no competing interests.

## Authors’ contributions

ELS collected some of the animals, performed the cytogenetic studies and drafted the manuscript. RSB performed the cytogenetic studies and drafted the manuscript. PPPM collected some of the animals, supervised the cytogenetic studies, helped draft the manuscript and revised the final text. All authors read and approved the final manuscript.
